# Letter to the editor regarding ‘Correlation between neutrophil-to-lymphocyte ratio and contrast-induced acute kidney injury and the establishment of machine-learning-based predictive models’

**DOI:** 10.1080/0886022X.2024.2307957

**Published:** 2024-01-24

**Authors:** Ning Zhuo, Gang Wang, Gang Wu

**Affiliations:** a Department of Nephrology, The Affiliated Suzhou Hospital of Nanjing Medical University, Suzhou Municipal Hospital, Suzhou, China; b Department of Rheumatology and Immunology, The Second Affiliated Hospital of Soochow University, Suzhou, China

Dear Editor,

We read with interest the recent publication by Zhou and colleagues on the correlation between neutrophil-to-lymphocyte ratio (NLR) and contrast-induced acute kidney injury and the establishment of machine-learning-based predictive models [1]. The authors found that a high NLR (>2.844) was an independent risk factor for CI-AKI (odds ratio = 2.304, *p* < 0.001). The area under the ROC curve (AUC) of the Naïve Bayes (NB) model was the largest (0.774), indicating that it had the best performance. NLR, serum creatinine concentration, fasting plasma glucose concentration, and use of β-blocker all accounted for a large proportion of the predictive performance of each model and were the four most important factors affecting the occurrence of CI-AKI [1]. We support and appreciate the authors’ work and agree with their conclusions, but have some concerns about some of the details in the article.

Firstly, drugs can have important effects on hematological parameters. Endogenous cortisol and catecholamines may be major drivers of NLR. High levels of cortisol are known to increase neutrophil counts while decreasing lymphocyte counts [2,3]. Similarly, endogenous catecholamines (e.g., epinephrine) can cause leukocytosis and lymphocytopenia. Cytokines and other hormones may also be involved. Therefore, confounding factors must be considered in order to assign the correct clinical significance to NLR alterations. Therefore, the effects of drugs represented by catecholamines and glucocorticoids should be considered in addition to diuretics, antiplatelet agents, anticoagulants, antibiotics, and β-blockers included in the article by zhou et al. [1].

Secondly, the time point of the article by Zhou et al. was studied in patients who underwent elective coronary angiography (CAG), and percutaneous coronary intervention (PCI) at the Department of Cardiology and elective vascular interventional surgery at the Department of Vascular Surgery, Ningbo NO.2 Hospital, Ningbo, China, from 1 January 2016 to 31 December 2020 [1]. Several studies have described the clinical characteristics of patients with Coronavirus disease 2019 (COVID-19), showing that NLR tends to be higher in critically ill patients [4,5]. Therefore, COVID-19 infection is one of the important diseases affecting NLR that cannot be ignored. The study by Buonacera and colleagues describes the main confounding factors affecting the NLR, including bacteremia and sepsis, active haematological disorders, HIV, acute myocardial infarction, stroke, pulmonary embolism, pneumonia, cancer, acute trauma, and emotional stress, in addition to the diseases mentioned in the article by Zhou et al. [1,4,5]. Therefore, if the authors could included the above indicators in their analyses, the results would have been more satisfactory.

Finally, NLR is an independent prognostic factor for mortality and morbidity in several diseases, and its normal threshold value is still under debate. A large retrospective case-control study implemented by Forget et al. [6] showed that the normal values of NLR values may range from 0.78 to 3.53 in a healthy and fit adult non-elderly population. A prospective, long-term population-based cohort study in the Rotterdam area of the Netherlands showed that, a mean NLR of 1.76, a 2.5% limit of 0.83 and a 97.5% limit of 3.92 in the general population [7]. In addition, the mean NLR was significantly higher in males (mean NLR of 1.88) than in females (mean NLR of 1.68), and was higher in older subjects >85 years of age (mean NLR of 2.13) than in subjects aged 45-54 years (mean NLR of 1.63) (*p* < 0.001) [7]. Given the above evidence, in the absence of standardized NLR thresholds, it may be challenging to apply the NLR as suggested by Zhou et al. [1].

In conclusion, before these issues are clarified, this study’s findings should be interpreted cautiously.
